# Preharvest UV-C Hormesis Induces Key Genes Associated With Homeostasis, Growth and Defense in Lettuce Inoculated With *Xanthomonas campestris* pv. *vitians*

**DOI:** 10.3389/fpls.2021.793989

**Published:** 2022-01-17

**Authors:** Amadou Sidibé, Marie Thérèse Charles, Jean-François Lucier, Yanqun Xu, Carole Beaulieu

**Affiliations:** ^1^Department of Biology, Université de Sherbrooke, Sherbrooke, QC, Canada; ^2^Saint-Jean-sur-Richelieu Research and Development Centre, Agriculture and Agri-Food Canada, Saint-Jean-sur-Richelieu, QC, Canada; ^3^College of Biosystems Engineering and Food Science, Zhejiang Key Laboratory for Agri-Food Processing, Zhejiang University, Hangzhou, China

**Keywords:** bacterial disease, cell homeostasis, defense mechanisms, eustress, leaf spot disease, lettuce, physiological processes, plant growth

## Abstract

Preharvest application of hormetic doses of ultraviolet-C (UV-C) generates beneficial effects in plants. In this study, within 1 week, four UV-C treatments of 0.4 kJ/m2 were applied to 3-week-old lettuce seedlings. The leaves were inoculated with a virulent strain of *Xanthomonas campestris* pv. *vitians* (*Xcv*) 48 h after the last UV-C application. The extent of the disease was tracked over time and a transcriptomic analysis was performed on lettuce leaf samples. Samples of lettuce leaves, from both control and treated groups, were taken at two different times corresponding to T2, 48 h after the last UV-C treatment and T3, 24 h after inoculation (i.e., 72 h after the last UV-C treatment). A significant decrease in disease severity between the UV-C treated lettuce and the control was observed on days 4, 8, and 14 after pathogen inoculation. Data from the transcriptomic study revealed, that in response to the effect of UV-C alone and/or UV-C + Xcv, a total of 3828 genes were differentially regulated with fold change (|log2-FC|) > 1.5 and false discovery rate (FDR) < 0.05. Among these, of the 2270 genes of known function 1556 were upregulated and 714 were downregulated. A total of 10 candidate genes were verified by qPCR and were generally consistent with the transcriptomic results. The differentially expressed genes observed in lettuce under the conditions of the present study were associated with 14 different biological processes in the plant. These genes are involved in a series of metabolic pathways associated with the ability of lettuce treated with hormetic doses of UV-C to resume normal growth and to defend themselves against potential stressors. The results indicate that the hormetic dose of UV-C applied preharvest on lettuce in this study, can be considered as an eustress that does not interfere with the ability of the treated plants to carry on a set of key physiological processes namely: homeostasis, growth and defense.

## Introduction

Plants live in a complex and constantly changing environment, where they continuously interact with biotic and abiotic factors (Foyer et al., 2016). These factors can be the cause of severe economic losses in edible plants such as lettuce, one of the most widely consumed leafy vegetables in the world (Moraes et al., 2018). The susceptibility of lettuce to certain diseases such as bacterial leaf spot (BLS), caused by *Xanthomonas campestris* pv. *vitians* (*Xcv*) can result in 100% yield losses (Lu and Raid, 2013). The bacterium can remain viable for months in buried plant debris or in surface irrigation water (Fayette et al., 2018) and penetrates leaves through stomata or wounds, resulting in bacterial clusters in the substomatal chamber (Bull et al., 2007). Typical symptoms of BLS begin as dark, oily looking spots and then cluster into large necrotic patches in warm, moist conditions (Bull et al., 2007) making the lettuce heads unsaleable. Confine Extra (mono and di-potassium salts of phosphorous acid) is the only chemical pesticide registered for the control of BLS in 2017 (Health Canada, 2019). To obtain the expected biological response, pesticides are used excessively and repeatedly, resulting in enormous costs and posing a serious threat to the environment and human health (Konatu and Jardim, 2018). Therefore, new and effective biological approaches to improve the control of BLS are urgently needed.

Recent scientific literature indicates increased interest in the use of biotic or abiotic agents as eustressors to improve plants’ functional quality and protect them against harmful levels of stressors. A biotic or abiotic eustressor is defined as a factor of biological, chemical or physical origin that can impact the physiological status of the plants in a beneficial way (Vázquez-Hernández et al., 2019). Very little is known about the cellular and molecular responses of plants to combined stimulation by a biotic disease-causing agent and an abiotic eustressor.

In response to biotic stresses, plants have developed a series of molecular pathways to adapt using proteins involved in cellular mechanisms as key players in the maintenance of cell homeostasis. Through regulation of physiological parameters, proteins participate directly in the response to biotic stresses giving rise to new plant phenotypes with peculiar characteristics (Feussner and Polle, 2015). The sequential effect of a certain combination of abiotic and biotic stresses may have a positive or negative impact on the plant’s response in terms of pathogen resistance and tolerance to abiotic stresses (Zhang and Sonnewald, 2017), depending on the level applied. For example, the effect of drought stress on tomato plant growth has led to increased resistance to *Botrytis cinerea* and *Oidium neolycopersici* (Achuo et al., 2006).

Research has shown that following applications of abiotic stresses such as heat stress, plants activate several molecular cascades involving kinases that regulate genes, transcription factors (TFs), microRNAs, reactive oxygen species (ROS) and Hsps (Muthusamy et al., 2017). Abiotic stresses result in the activation of numerous ion channels, signaling pathways of different hormonal cascades (ethylene, salicylic acid, abscisic acid, jasmonic acid) to overcome the adverse effects caused by the stress situation (Murcia et al., 2017). A recent study showed that proteometabolomic and physiological changes in algae due to ultraviolet-C (UV-C) doses of radiation are characterized by an increase in redox homeostasis, ROS production, protein damage and repair, avoidance elements, photosynthetic electron flux, carbon fixation, and C/N metabolism (Colina et al., 2020).

A complete understanding of metabolic and signaling pathways and their interactions during stress response is critical to enabling the development of stress-tolerant plants. The use of data from transcriptomics studies can provide comprehensive information on the interactions that occur in response to a specific stress (Bagati et al., 2018) such as UV-C. While in strawberries, studies have shown that treatment with UV-C has a significant impact on gene expression and leads to the overexpression of a set of genes required for effective defense in plant-pathogen interaction (Xu et al., 2019), the impact of UV-C on gene expression in lettuce is unknown.

Numerous studies have shown the beneficial effects of postharvest UV-C hormesis in several plant species (for a review, see Duarte-Sierra et al., 2019). When applied at the preharvest stage, the beneficial effects of UV-C hormesis have been shown in strawberry (Janisiewicz et al., 2016a,b; Xie et al., 2016; Xu et al., 2019), tomato (Valencia et al., 2017), and lettuce (Vàsquez et al., 2017; Sidibé et al., 2021). Among the beneficial effects of UV-C on lettuce, Sidibé et al. (2021) showed an increase in dry matter and resistance against *Xcv* with no negative impact on yield. The work of Sidibé et al. (2021) suggests that the lettuce plant is able to integrate the negative effects of UV-C-induced oxidative stress signals, return to a state of homeostasis and pursue normal growth while mounting an effective defense fight against BLS. Based on these premises, using transcriptomics in the present study, we propose a hypothetical model describing the regulatory mechanisms that may underlie the beneficial effects induced by preharvest hormetic doses of UV-C radiation on lettuce.

## Materials and Methods

### Plant Material and Inoculation

The lettuce cultivar ‘Parris Island Cos’, which is known to be susceptible to the pathogen *Xanthomonas campestris* pv. *vitians*-B07-007 (*Xcv*) (Nicolas et al., 2018), was used in this study. Seeding was carried out in a 98-well tray in a germinator for 2 weeks, and seedlings were then transferred to growth chambers as described by Sidibé et al. (2021). The plants were watered daily, alternating between water and a nutrient solution containing 200 ppm nitrogen, 71 ppm potassium and 200 ppm phosphorus. Inoculation was done with a virulent *Xcv* strain B07-007 isolated from lettuce fields in Montérégie (QC, Canada).

The inoculation conditions were described by Nicolas et al. (2018). Briefly, *Xcv* B07-007 was recovered in sterile distilled water after 48 h culture on YDC agar medium at a final concentration of 10^8^ colony forming units (CFU) per mL and then sprayed up to runoff on the adaxial and abaxial surfaces of the leaves of 18 lettuce plants, 9 control, and 9 UV-C treated (Supplementary Figure 1) using a manual sprayer.

### Ultraviolet-C Treatments

Ultraviolet-C treatments began 3 weeks after sowing, corresponding to the transplanting stage of lettuce in the field as described by Sidibé et al. (2021). The growth chambers (Conviron, PGV40, MB, Canada) were modified with a set of three UV-C lamps (254 nm, 160 W, Clean Light Inc., Vineland Station, ON, Canada). The lamps were placed at 80 cm over the tops of the plants. During treatment, the intensity of the lamps was 1.1 mW/cm^2^ measured with a radiometer (ML1400A, Miltec UV, Stevensville, MD, United States) equipped with a SEL240 # 6090 sensor (ML1400A, Miltec UV). The time required to administer the treatment dose of 0.4 kJ/m^2^ was 1 min. Four UV-C doses of 0.4 kJ/m^2^ (Supplementary Figure 1) at intervals of 48 h between the first three doses and 72 h for the last dose was applied (Nicolas et al., 2020). In total, lettuce plants in the treated group received 1.6 kJ/m^2^ over a 1-week period. Forty-eight hours after the last UV-C treatment, lettuce was inoculated with *Xcv* B07-007 as described above and in Supplementary Figure 1.

### Evaluation of the Effect of Ultraviolet-C Treatments on Bacterial Leaf Spot Symptom Expression

The method of Nicolas et al. (2018) was used to assess BLS symptom development. The severity of the disease is the proportion of the foliar surface with symptoms of disease (Nicolas et al., 2018). The area under the disease progress curve (AUDPC) was calculated using equation (1) for trapezoidal integration of disease incidence as described by Shaner and Finney (1977).


(1) $$A\,U\,D\,P\,C=\sum_{i=1}^{n}\left(\frac{Y_{i+n\,1}+Y_{i}}{2}\right)\,\left(X_{i+1}+X_{i}\right)$$

where Y is the disease severity (per unit) at the *i*th observation, X*i* is the time (days) at the *i*th observation, and n is the total number of observations.

This experiment was repeated three times.

### RNA Extraction, Transcriptomic and qPCR Analysis

A transcriptomic study was carried on lettuces in three independent completely randomized experiments (E1, E2, E3), each designed with three replicates of three experimental units (Supplementary Table 1 and Supplementary Figure 1). Fully extended lettuce leaves without the main vein from each replicate were sampled at two different times, T2 and T3 (Supplementary Figure 1). For each experiment, a total of 54 plants were randomly selected after transplantation and acclimatization. At this time, the plants were separated into two groups: 27 plants received the UV-C treatment and 27 plants served as control. In each group (UV-treated and control), 48 h after the last UV treatment (T2), nine plants (3 rep × 3EU) were randomly sampled as UV-48 h and C-48 h. Also at T2 inoculation was performed on nine plants from each group but there was no sampling. Later, at 24 h post-inoculation (T3), nine plants from each inoculated group were sampled to provide UVi-24 h and Ci-24 h. At the same time (T3) as an un-inoculated reference set, the nine remaining plants from each group were sampled to provide UV-72 h and C-72 h. Once the leaf samples were severed from the plants, they were immediately flash-frozen in liquid nitrogen and stored at –80°C until RNA extraction.

The total RNA of the leaves (0.1 g) was extracted using a Qiagen total RNA isolation system (RNeasy Plant Mini Kit, 74904, Qiagen, Valencia, CA, United States) according to the manufacturer’s protocol. Any contaminating genomic DNA that may have been extracted along with the total RNA was removed using the RNase free DNase kit according to the instructions provided by the manufacturer (Qiagen, Germantown, United States). The verification of RNA sample purity was performed by the Bioanalyzer Agilent 2100 system using the Nano 6000 Assay RNA kit (Bioanalyzer 2100, CA, United States). This system provides the quantity and size of the RNA strands and the evaluation of the purity of the RNA samples. The RNA integrity number (RIN) minimum accepted was 5 and the mean RIN of the samples obtained was 8.

The transcriptomic analysis was performed by Genome Quebec (Canada). The cDNA libraries were built up from RNA samples by paired-end (PE) points sequencing following the Illumina Next-Generation Sequencing (NGS) protocol. The sequencing of the PE (2 × 100 bp) was performed on the Illumina HiSeq 2000 platform at a rate of 200 million sequences per line with an error rate < 0.1% (Illumina, San Diego, CA, United States). The RNA-seq library reads were trimmed using Trimmomatic 0.36 and aligned on lettuce reference genome Lsat_Salinas_v7 (GCF_002870075.1) using the STAR aligner v2.5.3a^1^.

The expression profile of 10 randomly selected lettuce genes from RNA-seq data was validated using qPCR. List of primers used in this study is presented in Supplementary Table 2. For each gene, three biological replicates with two technical replicates were used. The EXP45 gene was chosen as reference genes (Borowski et al., 2014). Prior to -qPCR, cDNA biosynthesis was performed from 5 μg of each RNA sample using the Maxima Mix reverse transcription kit (Thermo Scientific) according to the manufacturer’s instructions.

qPCR reactions were performed in a final volume of 20 μl and contained 10 μl of the ROX qPCR Master Mix from Thermo Fisher Scientific, 0.5 μM of each primer, and 2 μl of cDNA, equivalent to 20 ng of total RNA. PCR thermocycling conditions were set at 95°C for 3 min, 35 cycles at 95°C for 5 s, 60°C for 30 s, in a QuantStudioTM 3. Specificity of amplification as well as the absence of primer dimers was confirmed by melting curve analysis at the end of each reaction.

To correct for technical variations in reverse transcription and qPCR reactions as well as expression data for biological variations, these were normalized to the geometric mean of the EXP45 reference gene. The fold change (±SD) is expressed as treatment versus control is calculated by the 2-ΔΔCt method (Livak and Schmittgen, 2001).

### Statistical Analysis

The experiment consisted of three independent repeats in a completely randomized design with three replicates of three experimental units, an experimental unit being 1 lettuce plant in a pot. For each experiment, the lettuce seedlings were randomly assigned to two separate groups 3 weeks after sowing. One group received the UV-C treatment according to the different modalities described above (see section “Ultraviolet-C Treatments”). Another group received no treatment and served as the control group. Symptoms of BLS data were subjected to a multi-factor analysis of variance (ANOVA) with interactions on R (v3.6.2), and Python was used to create the time-based disease assessment curve. The statistical procedures for the ANOVA and the time-based disease assessment curve can be found at https://bitbucket.org/asidibe2011/bls_data_analysis and https://bitbucket.org/asidibe2011/python_curve, respectively. RNA-Seq differential transcript expression levels were computed using Cufflinks v2.2.1, and differential relative gene expression analyses were computed using DESEQ and EDGER from the Bioconductor Rtools suite v3.5.0. Heatmaps were generated with R (v3.6.2) using the “pheatmap” package to show the relative expression of genes in the different samples. The procedures are available at https://bitbucket.org/asidibe2011/heatmaps/src/66fad937f2b5?at=master. Transcripts indicating a fold change ≥ 1.5 with FDR < 0.05 were confirmed to be differentially expressed. It should also be noted that the selection of the genes presented in the results is based on their function as described in the literature (Supplementary Table 3).

## Results

### Increased Resistance in Ultraviolet-C-Treated Lettuce to *Xanthomonas campestris* pv. *vitians*

**FIGURE 1:**
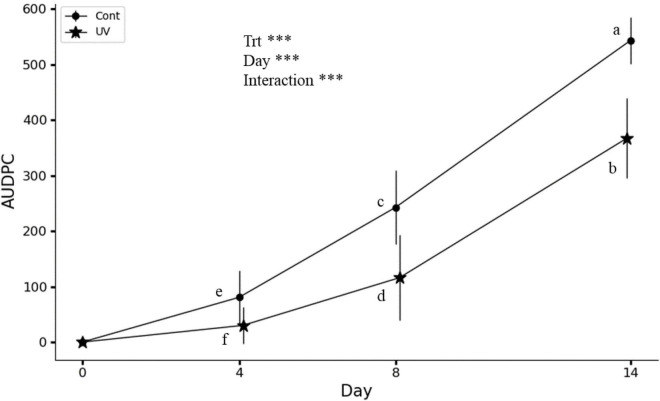
Effect of UV-C treatments on AUDPC (area under the disease progress curve). Treatments (Trt): Cont (control), UV (1.6 kJ/m^2^). Two-way ANOVA indicates a significant effect of treatments, assessment days (0, 4, 8, and 14) for BLS (bacterial leaf spot) and their interaction. AUDPC means are statistically different at *P* < 0.05 according to the Duncan’s multiple range test, *n* = 9, ^***^P < 0.001. Different letters indicate a significant difference between treatments at the same day or on different days (4, 8, and 14). The vertical bars show the standard deviation.

Significant differences were observed between UV-C treated lettuce and controls in terms of *Xcv* symptom expression on days 4, 8, and 14 after inoculation (Figure 1). On day 4, UV-C treated lettuce leaves showed very few apparent symptoms, resulting in an AUDPC value close to 0, while on the leaves of the control lettuce, some dark and oily spots were observed, resulting in an AUDPC value of around 80. From day 8 on, the symptoms appearing on the lettuces were small necrotic spots, evolving to larger spots by day 14. This evolution was weak in UV-C treated lettuces compared to controls. As a result, AUDPC was significantly smaller in the treated group, with UV-C treatment resulting in a 62, 52, and 30% reduction in symptoms at 4, 8, and 14 days after *Xcv* inoculation, respectively, indicating an increase in AUDPC over time. However, this increase was significantly higher in control lettuces.

### Differential Relative Expression of the RNA Sequences

RNA sequencing of the 54 samples from three biological replicas yielded a total gross readings of around 3 billion 94 million. Net readings of 3 billion 29 million, or around 97.88% of gross readings, were associated with the reference genome of *Lactuca sativa* L. (Supplementary Table 4). A total of 46,150 transcripts were identified. In UV-C treated lettuces, UV-48 h and UV-72 h and their controls C-48 h and C-72 h, respectively, 31538, 31371, 31370, and 31000 transcripts were detected, while in inoculated UV-C treated lettuces and the corresponding inoculated controls, 30930 and 31231 transcripts were detected, respectively (Supplementary Figure 2). The number of differentially expressed genes were 265, 2329, 2683, 2399, and 2653 in UV-48 h, UV-72 h, C-72 h, Ci-24 h and UVi-24 h respectively (Table 1 and Supplementary Figure 3).

**TABLE 1:** Overall number of unique genes per sample.

Samples^1^	Number of genes	Number of genes specific to the sample
UV-48 h	265	44 (20-up, 24-down regulated)
C-72 h	2683	422 (161-up, 261-down regulated)
UV-72 h	2329	257 (117-up, 140-down regulated)
Ci-24 h	2399	107 (107-up regulated)
UVi-24 h	2653	238 (238-up regulated)
Unique genes	3828	

*^1^Sample genes result from their comparison with the C-48 h sample as control. C-48 h, control corresponding to treated 48 h after UV. UV-48 h, UV after 48 h treatment; C-72 h, control corresponding to treated 72 h after UV; UV-72 h, UV after 72 h treatment; Ci-24 h: Control 24 h after inoculation; UVi-24 h, UV 24 h after inoculation. The genes were filtered out with a threshold |log2-FC| > 1.5 and FDR < 0.05.*

### Overall Functions of Genes Affected by Preharvest Ultraviolet-C and *Xcv*

**FIGURE 2:**
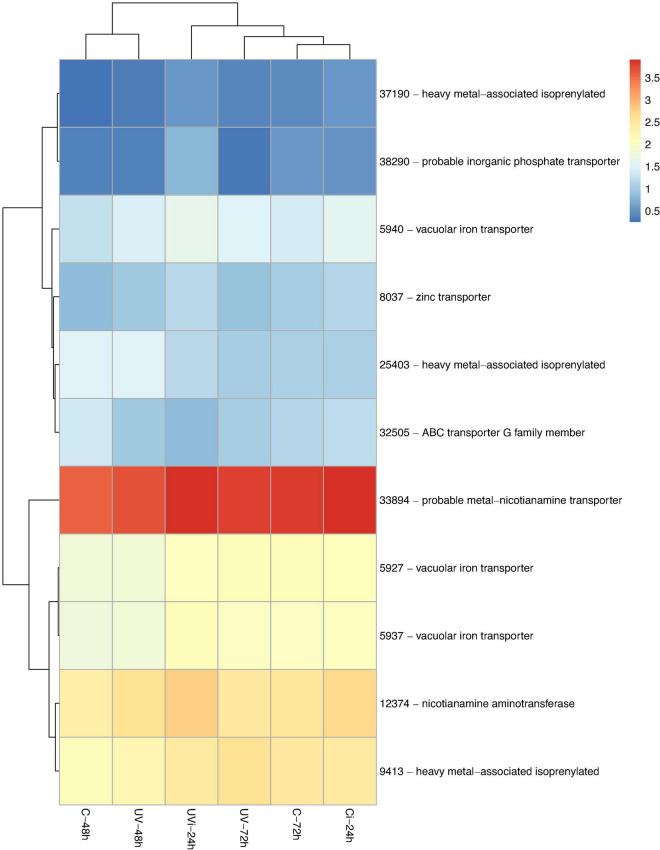
Genes related to metal transport differentially expressed in response to UV and *Xcv* inoculation. Test of hierarchical DEG (HCA) groupings. The colors reflect the level of relative gene expression. The columns and rows represent the samples and ID_genes associated with the name/function of the corresponding proteins that were grouped according to their relative expression profile, respectively. C-48 h, control corresponding to treated 48 h after UV; UV-48 h, UV after 48 h treatment; C-72 h, control corresponding to treated 72 h after UV; UV-72 h, UV after 72 h treatment; Ci-24 h, control 24 h after inoculation; UVi-24h, UV 24 h after inoculation. The genes were filtered out with a threshold |log2-FC| > 1.5 and FDR < 0.05.

**FIGURE 3:**
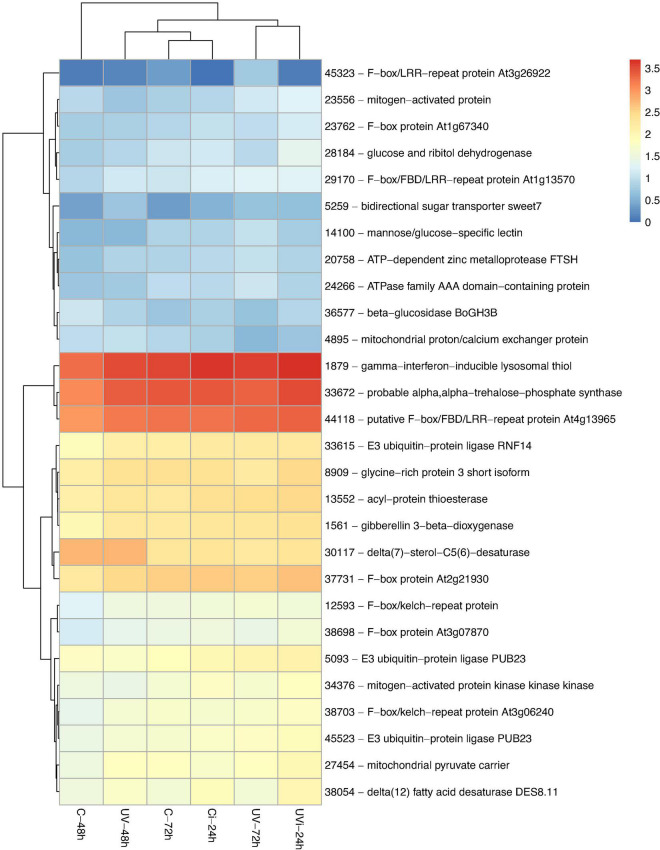
Primary metabolism-related genes differentially expressed in response to the influence of UV and *Xcv* inoculation. Test of hierarchical DEG (HCA) groupings. The colors reflect the level of relative gene expression. The columns and rows represent the samples and ID_genes associated with the name/function of the corresponding protein that were grouped according to their relative expression profile, respectively. C-48 h, control corresponding to treated 48 h after UV; UV-48 h, UV after 48 h treatment; C-72 h, control corresponding to treated 72 h after UV; UV-72 h, UV after 72 h treatment; Ci-24 h, control 24 h after inoculation; UVi-24 h, UV 24 h after inoculation. The genes were filtered out with a threshold |log2-FC| > 1.5 and FDR < 0.05.

**FIGURE 4:**
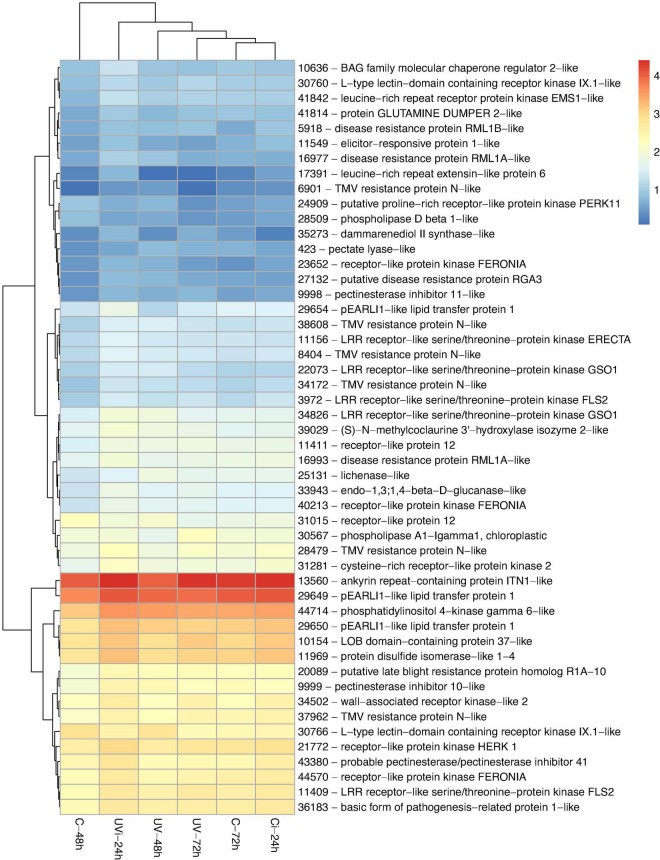
Genes related to defense mechanisms differentially expressed in response to UV and *Xcv* inoculation. Test of hierarchical DEG (HCA) groupings. The colors reflect the level of relative gene expression. The columns and rows represent the samples and ID_genes associated with the name/function of the corresponding protein that were grouped according to their relative expression profile, respectively. C-48 h, control corresponding to treated 48 h after UV; UV-48 h, UV after 48 h treatment; C-72 h, control corresponding to treated 72 h after UV; UV-72 h, UV after 72 h treatment; Ci-24 h, control 24 h after inoculation; UVi-24 h, UV 24 h after inoculation. The genes were filtered out with a threshold |log2-FC| > 1.5 and FDR < 0.05.

By comparing the identified transcripts of the samples from the different groups of lettuce with the C-48 h, 3828 differentially expressed transcripts with |log2-FC| > 1.5 and false discovery rate (FDR) < 0.05 were detected. The functions of 59% of these transcripts (2270) have been defined (Supplementary Table 5). Among these 2270 genes, some were unique to the different treatments: 44 genes belonged to UV-48 h, 257 genes to UV-72 h, 422 genes to C-72 h, 107 genes to Ci-24 h, and 238 genes to UVi-24 h. These differentially expressed genes are associated with 14 biological functions including stress and antioxidant response (Table 2 and Figure 2); cell homeostasis and cell cycle (Table 3); metal transport (Figure 2); cell morphology and plant growth (Table 4); primary metabolism (Figure 3 and Supplementary Table 4); cell wall (Table 5); defense mechanisms (Figure 4); chloroplasts, phytohormones and other phenylpropanoids (Table 6). These differentially expressed genes can therefore explain the modification of the lettuce transcriptome following the effect of UV-C treatments followed by *Xcv* inoculation at three levels: homeostasis state, resumption of normal growth and activation of defense mechanisms.

**TABLE 2:** Relative expression of stress and antioxidant response genes.

Samples	Gene ID	Log2-FC	Protein name
C-48 h*UV-72 h	7750	1.735	Probable serine/threonine-protein kinase PBL22
	25745	1.582	Probable protein phosphatase 2C 39
	40915	1.531	Probable WRKY transcription factor 40
	44001	1.525	BURP domain protein USPL1
	33752	–1.709	Peptidyl-prolyl cis-trans isomerase-like
C-48 h*UVi-24 h	22470	2.475	Homogentisate phytyltransferase 1, chloroplastic-like
	31324	2.24	Probable carboxylesterase 18
	45305	2.129	CBL-interacting serine/threonine-protein kinase 7-like
	25732	1.994	Probable WRKY transcription factor 70
	33507	1.789	Protein ABHD18
	25564	1.788	Sulfate transporter 1.3
	44623	1.706	Peroxidase N1-like
	1939	1.659	Epidermis-specific secreted glycoprotein EP1-like
	4132	1.624	Monothiol glutaredoxin-S2-like
	17850	1.614	Calmodulin-binding transcription activator 3-like
	36553	1.61	Heme oxygenase 1, chloroplastic-like
	23563	1.523	Probable WRKY transcription factor 31
	8354	1.505	Receptor-like cytoplasmic kinase 176

*The genes were filtered out with a threshold |log2-FC| > 1.5 and FDR < 0.05. C-48 h, control corresponding to treated 48 h after UV. UV-48 h, UV after 48 h treatment; UV-72 h, UV after 72 h treatment; UVi-24 h, UV 24 h after inoculation.*

**TABLE 3:** Relative expression of cell homeostasis and cell cycle arrest genes.

Processes	Samples	Gene ID	Log2-FC	Protein name
Homeostasis	C-48 h*UV-48 h	19989	–1.957	Calcium-binding protein PBP1-like
	C-48 h*UV-72 h	27642	1.761	Calcium-binding protein CML38-like
	C-48 h*UVi-24 h	39875	1.838	Inorganic phosphate transporter 1–4-like
		4352	1.776	AAA-ATPase At5g57480-like
		11830	1.604	Protein EARLY FLOWERING 3
		33035	1.557	Calcium-dependent protein kinase 7-like
Cell cycle arrest	C-48 h*UV-48 h	38678	–1.506	U-box domain-containing protein 1-like
	C-48 h*UV-72 h	7029	1.502	U-box domain-containing protein 19-like
		14274	–1.717	Cyclin-D5-3-like
	C-48 h*UVi-24 h	9509	1.624	Cyclin-dependent kinase inhibitor 3-like
		44951	1.606	U-box domain-containing protein 13-like
		26329	1.539	U-box domain-containing protein 29-like

*The genes were filtered out with a threshold |log2-FC| > 1.5 and FDR < 0.05. C4–8 h, control corresponding to treated 48 h after UV. UV-48 h, UV after 48 h treatment; UV-72 h, UV after 72 h treatment; UVi-24 h, UV 24 h after inoculation.*

**TABLE 4:** Relative expression of cell morphology and growth genes.

Processes	Samples	Gene ID	Log2-FC	Protein name
Morphology	C-48 h*UV-48 h	38675	2.246	Zinc finger protein CONSTANS
	C-48 h*UV-72 h	45199	2.025	Zinc finger protein ZAT12
		20999	–1.552	Very-long-chain enoyl-CoA reductase
		41210	–1.636	Zinc finger MYM-type protein 1
	C-48 h*UVi-24 h	35244	1.593	Cell number regulator 7
Growth	C-48 h*UV-72 h	29487	2.222	Protein DOG1
		44813	1.725	Splicing factor U2af small subunit B
		32006	1.66	NAC domain-containing protein
		23793	1.62	Scarecrow-like protein 15
		25468	1.615	Phosphoprotein ECPP44
		37511	1.584	Scarecrow-like protein 8
		45340	–1.775	Ribonuclease H2 subunit B
		43958	–4.114	Protein JINGUBANG
	C-48 h*UV-i24 h	20982	2.775	RING-H2 finger protein ATL70
		40351	2.107	Inositol 2-dehydrogenase 2
		6073	2.049	Serine/threonine-protein kinase
		32107	1.788	RING-H2 finger protein
		9835	1.736	Serine/threonine-protein kinase
		2883	1.707	Upstream activation factor
		19997	1.698	Nuclear transcription factor Y
		32907	1.691	LanC-like protein GCL1
		29278	1.686	Probable mediator of RNA
		20239	1.624	Serine/threonine-protein kinase
		16308	1.602	Nucleolar GTP-binding protein
		15163	1.578	Protein DOG1
		25756	1.572	Protein cup-shaped cotyledon
		26522	1.56	NAC transcription factor
		33732	1.552	Myb-related protein 308
		18254	1.55	RING-H2 finger protein ATL54
		35967	1.538	BES1/BZR1 homolog protein
		32104	1.523	RING-H2 finger protein ATL78

*The genes were filtered out with a threshold |log2-FC| > 1.5 and FDR < 0.05. C-48 h, control corresponding to treated 48 h after UV; UV-48 h, UV after 48 h treatment; UV-72 h, UV after 72 h treatment; UVi-24 h, UV 24 h after inoculation.*

**TABLE 5:** Relative expression of cell wall genes.

Samples	Gene ID	Log2-FC	Protein name
C-48 h*UV-72 h	4681	1.704	WAT1-related protein At2g39510-like
	23001	–1.518	Expansin-A15-like
	5596	–1.524	UPF0481 protein At3g47200-like
	23655	–1.57	Putative receptor-like protein kinase At5g39000
	39520	–1.618	G2/mitotic-specific cyclin C13-1-like
	32919	–1.746	Classical arabinogalactan protein 2-like
	17410	–1.919	Extensin-3-like
C-48 h*UVi-24 h	14233	3.193	Putative receptor protein kinase ZmPK1
	6251	2.61	Extensin-2-like
	5010	2.189	Probable receptor-like protein kinase At5g38990
	23417	2.186	Berberine bridge enzyme-like 8
	27222	2.095	Clavaminate synthase-like protein At3g21360
	33528	1.748	UPF0481 protein At3g47200-like
	15855	1.663	Probable serine/threonine-protein kinase PIX13
	8086	1.662	Extensin-1
	38017	1.641	Heparanase-like protein 1
	11861	1.613	Probable receptor-like protein kinase At5g20050
	37407	1.608	Formin-like protein 4
	35108	1.583	Protein LURP-one-related 10-like

*The genes were filtered out with a threshold |log2-FC| > 1.5 and FDR < 0.05. C-48h, control corresponding to treated 48 h after UV; UV-48 h, UV after 48 h treatment; UV-72h, UV after 72 h treatment; UVi-24 h, UV 24 h after inoculation.*

**TABLE 6:** Relative expression of phytohormones, chloroplast, and phenylpropanoids biosynthesis genes.

Processes	Samples	Gene ID	Log2-FC	Protein name
Phytohormones	C-48 h*UV-72 h	1159	2.076	Ethylene-responsive transcription factor
		27723	1.761	Geraniol 8-hydroxylase
		16982	–1.792	Glutamate receptor 3.6
	C-48 h*UVi-24 h	23265	1.8	Ethylene-responsive transcription factor
		24354	1.771	Glutamate receptor 2.8
		45812	1.748	Auxin-responsive protein
		34865	1.636	Glutamate receptor 1.3
		16974	1.61	Glutamate receptor 3.6
		17418	1.573	Ethylene-responsive transcription
		2086	1.546	Glutamate receptor 1.2
Chloroplast	C-48 h*UV-72 h	22355	–1.518	Cytochrome P450 89A2
		21432	–1.706	Cytochrome P450 71A4
	C-48 h*UVi-24 h	24501	1.89	Cytochrome P450 94B3
		10522	1.739	Cytochrome P450 71A6
		40902	1.72	Clavaminate synthase-like protein
Other phenylpropanoids	C-48 h*UV-72 h	29845	2.179	β-galactosidase 15
		11168	1.589	*S*-norcoclaurine synthase 2
		23316	1.531	β-amyrin 28-oxidase
		14651	1.503	Serine carboxypeptidase
		46017	–1.529	Palmitoyl-acyl carrier protein
		36801	–2.339	Hydroxycinnamoyltransferase
		46011	–2.698	Palmitoyl-acyl carrier protein
	C-48 h*UVi-24 h	13938	1.967	Vinorine synthase
		768	1.899	Vitellogenin-2
		38075	1.778	Isoflavone 2′-hydroxylase
		36806	1.593	Putrescine hydroxycinnamoyltransferase
		6395	1.52	Protein SRC2

*The genes were filtered out with a threshold |log2-FC| > 1.5 and FDR < 0.05. C-48 h, control corresponding to treated 48 h after UV; UV-48 h, UV after 48 h treatment; UV-72 h, UV after 72 h treatment; UVi-24 h, UV 24 h after inoculation.*

#### Genes Linked to Homeostasis State

Table 2 and Figure 2 show that the combined effect of UV-C and inoculation resulted in the relative overexpression of 13 genes, including those of the WRKY TFs, while the single effect of UV-C 72 h after treatment resulted in the relative overexpression of four genes and the downregulation of a single gene. At the level of genes associated with WRKY TFs and upregulated, there was only one gene (40915) induced by UV-C and two genes (25732 and 23563) induced by the combined effect of UV-C and inoculation. In addition, the combined effect of UV-C and inoculation resulted in positive regulation of the genes 22470, 44623, 31324, and 1939 associated with homogentisate phytyltransferase (HPT), peroxidase N1-like, probable carboxylesterase and epidermis-specific secreted glycoprotein (EP1), respectively.

A total of six genes (19989, 27642, 39875, 4352, 11830, 33035) linked to cell homeostasis calcium-binding protein PBP1-like, calcium-binding protein CML38-like, inorganic phosphate transporter 1-4-like, AAA-ATPase At5g57480-like, protein early flowering 3 and calcium-dependent protein kinase 7-like were differentially expressed (Table 3). The differential expression of these genes is due to the combined effect of UV-C and *Xcv*, with the exception of the genes encoding calcium-binding protein PBP1-like, calcium-binding protein CML38-like, inorganic phosphate transporter 1-4-like, which are due respectively to the effect of UV-C 48 h and 72 h after the last treatment. All these genes were upregulated with the exception of gene 19989 encoding a calcium-binding protein PBP1-like.

The gene 27642 linked to the calcium binding protein CML38-like (calmodulin-like proteins) and the gene 33035 linked to the calcium-dependent protein kinase 7-like were positively regulated in samples UV-72h and UVi-24h, respectively. In the UV-48h samples, gene 38678, associated with the U-box domain-containing protein 1-like, was observed to be downregulated. In the UV-72h samples, the genes (14274, 7029) linked to cyclin D5-3-like and 19-like protein containing the U-box domain were downregulated and upregulated, respectively.

A treatment-dependent regulation of three heavy-metal-binding protein biosynthesis genes (25403, 37190, and 9413) was observed (Figure 2). Genes 25403 and 9413 are respectively downexpressed and overexpressed in response to UV-72h, while lettuce in response to UVi-24 h resulted in positive regulation of gene 37190. In addition, UVi-24 h caused the relative overexpression of three genes (5927, 5937, and 5940) linked to the vacuolar iron transporter1 (VIT1) and one gene (12374) linked to nicotianamine aminotransferase A-like (NA).

#### Genes Linked to Resumption of Normal Growth

Among the genes involved in cell morphology, five were differentially expressed (Table 4) in UVi-24 h samples, one gene, 35244, which is associated with the cell number regulator 7-like (CNR), is overexpressed. In samples UV-48 h and UV-72 h, a positive regulation is observed with gene 38675, associated with zinc finger protein, Constans-like 10-like, and gene 45199, associated with zinc finger protein ZAT12-like, respectively.

Table 4 also indicates the upregulation of four genes (18254, 20982, 32107, and 32104) associated with ATL (RING-H2 finger protein) and of gene 35967 linked to BRI1-EMS suppressor1/brassinazole-resistant1 (BES1/BZR1) homolog protein 4-like in response to the effect of UV-C and *Xcv* (Table 2). The effect of UV-C alone 72 h after treatment resulted in the upregulation of gene 25468 linked to phosphoprotein ECPP44-like.

Concerning photosynthesis, gene 7707 associated with ruBisco (ribulose-1,5-bisphosphate carboxylase/oxygenase) was positively regulated following UV-C 48 and 72 h after the last treatment and also in response to the combined effect of UV-C and *Xcv* with respective FDR of 0.006, 1.4E-13, 7.2E-14 and log2-FC of 1.42, 2.84, and 2.96. Similarly, gene 35073 linked to bisphosphoglycerate was overexpressed by the combined effect of UV-C and *Xcv*, with a FDR of 1.5E-14 and log2-FC of 0.7.

The heatmap presented in Figure 3 show the relative expression of the genes mainly involved in primary metabolism. They include a group of five highly induced genes (38054, 1879, 33672, 28184, and 23004) encoding delta (12) fatty acid desaturase (DES8.11), gamma-interferon-inducible lysosomal thiol reductase, probable alpha alpha-trehalose phosphate synthase (TPS10) and glucose ribitol dehydrogenase-like. There were also two groups of moderately expressed genes linked to the F-box family. Of the F-box family, the gene associated with the repeat protein F-box/LRR At3g26922 is strongly overexpressed in response to UV-C, 72 h after the last treatment, with a fold change > 3 (Supplementary Table 5).

Several genes in Figure 3 and Table 3 are involved in protein ubiquitination. These are the genes associated with E3 ubiquitin-protein ligase PUB23-like (gene 45523), E3 ubiquitin-protein ligase PUB23-like (gene 5093), and E3 ubiquitin-protein ligase RNF14-like (gene 33615), with respective fold changes of 1.863, 1.539, and 1.503 (Figure 3 and Supplementary Table 5). In the treated and inoculated lettuce, three upregulated genes (9509, 44951, and 26329) were differentially expressed: cyclin-dependent kinase inhibitor 3-like, U-box domain-containing protein 13-like and U-box domain-containing protein 29-like. Two protein-associated genes (20758 and 24266) of the protease subfamily, namely the ATP-dependent zinc metalloprotease FtsH 6, located in the chloroplast, and At1g05910-like, a member of the ATPase family AAA (ATPase Associated with various cellular Activities), were positively regulated, with a respective fold change of 1.6 and 1.5 (Figure 3 and Supplementary Table 5) in response to the UV-C effect 72 h after treatment.

Our results indicate a positive upregulation of two genes (34376 and 23556) linked to mitogen-activated protein kinase 18-like, one gene (27454) related to mitochondrial pyruvate carrier (Figure 3 and Supplementary Table 5), and one gene (41814) linked to protein glutamine dumper 2-like (Figure 4) following the combined effect of UV-C and the pathogen *Xcv*.

In UV-72h samples, a single gene 4681 linked to WAT1-related protein At2g39510-like was overexpressed. However, in response to the combined effect of UV-C and inoculation, all 12 genes observed in Table 5 (14233, 6251, 5010, 23417, 27222, 33528, 15855, 8086, 38017, 11861, 37407, and 35108) were positively regulated and were associated with 12 different cell wall-related proteins. Among the proteins positively regulated in response to the combined effect of UV-C and inoculation are two clavaminate synthase-like proteins (Tables 2, 3). The combined effect of UV-C and inoculation also resulted in the relative overexpression of five genes (Figure 4 and Tables 4, 5) coding for proteins related to the maintenance of the cell wall: BES1/BZR1 homolog protein, leucine-rich repeat extensin protein (LRX), leucine-rich repeat receptor protein kinase and two extensin proteins.

#### Genes Linked to Activation of Defense Mechanisms

Our results (Figure 4) show a relative overexpression of five genes (11156, 3972, 11409, 34826, and 22073) linked to the receptor-like kinase (RLK) family protein biosynthesis and one ankyrin repeat-containing protein ITN1-like following the combined effect of UV-C and *Xcv* inoculation. The coding sequence of gene 28479, a precursor of the CRK proteins, was positively regulated in response to the combined effect of UV-C and inoculation.

As indicated in Figure 4, positive regulation was observed in response to the effect of UV-C and inoculation of three *AZI1* genes (29649, 29650, and 29654), gene 10636, which is associated with the biosynthesis of Bcl-2-associated athanogene (BAG) family, and gene 36183, which is associated with the biosynthesis of PR-1 (Pathogenesis Related) proteins. The pEARLI1-like lipid transfer protein 1 biosynthesis genes are known as AZI1.

The effect of UV-C alone 72 h after the last treatment resulted in downregulation of gene 31015 involved in the biosynthesis of receptor-like proteins (RLPs) and gene 30766 associated with L-type lectin receptor kinase (LECRKs). However, the combined effect of UV-C and inoculation resulted in the positive regulation of five RLP genes (11411, 40213, 23652, 44570, and 21772), one LECRKs gene (30760), and one wall-associated receptor kinase-like 2 (WAK) protein biosynthesis gene (34502) (Figure 2).

The data from Figure 4 show relative overexpression in response to UVi-24h of 9 genes (6901, 38608, 28479, 34172, 37962, 8404, 20089, 17391, and 41842). Gene 20089 is a presumed homologue of the mildew resistance protein R1A-10, and genes 17391 and 41842 are associated with the biosynthesis of proteins of the leucine-rich repeated extensin (LRX) family. With respect to the cell wall, the effect of UVi-24 h resulted in the relative overexpression of two genes (9999 and 9998) associated with the inhibition of pectin methylesterase proteins (PME) and three genes (43380, 9999, and 9998) linked to the biosynthesis of cell wall proteins (CWPs) and pectin. Gene 33943, a precursor of β-1,3:1,4-Glucan, and gene 25131, associated with glycan metabolism, are highly overexpressed as a result of UV-C and inoculation.

Figure 4 also shows positive regulation of four other disease resistance genes. The relative overexpression of genes 16977, 16993, and 27132, which are related to disease resistance proteins RML1A-like, RML1B-like and RGA3, is due to the combined effect of UV-C and inoculation with *Xcv*. The upregulation of gene 5918 associated with disease resistance is due solely to the effect of UV-C 72 h after the last treatment. Genes 24266 and 20758, linked to the AAA ATPase family (Figure 3) overexpressed in response to the effect of UV-C, are also involved in plant defense.

The results presented in Table 6 indicate that two genes (21432 and 22355) associated with cytochrome P450 were downexpressed in UV-72h. However, the combined effect of UV-C and inoculation with *Xcv* resulted in a positive regulation of two other genes (10522 and 24501) linked to cytochrome P450 and two genes (40902 and 27222) linked to clavaminate synthase-like protein.

Our study showed 10 differentially expressed hormone-related genes (Tables 4, 6). The effect of UV light 72 h after the last treatment resulted in relative overexpression of gene 1159, which is associated with the ethylene-responsive transcription factor ERF017-like. In addition, the combined effect of UV-C and inoculation resulted in the relative overexpression of two genes (23265 and 17418) involved in the biosynthesis of ERF family proteins and of four genes (24354, 34865, 16974, 2086) associated with the glutamate receptor GLRs.

Table 6 shows the relative overexpression of five genes (29845, 11168, 23316, 14651, and 27723) in UV-72h samples. These genes are related to biosynthesis of several enzymes, namely, β-galactosidase 15-like, *S*-norcoclaurine synthase 2-like, β-amyrin 28-oxidase-like, serine carboxypeptidase-like and geraniol 8-hydroxylase-like (G8H).

Table 6 show that the combined effect of UV-C and inoculation with *Xcv* resulted in the positive regulation of six genes (13938, 768, 38075, 36806, 6395, and 39029) associated with the biosynthesis of vinorin synthase-like, vitellogenin-2-like, isoflavone 2′-hydroxylase-like, putrescine hydroxycinnamoyltransferase 1-like (PHT), protein SRC2-like, and (*S*)-*N*-methylcoclaurine 3′-hydroxylase isozyme (CYP80B1). Two other genes (16310 and 25705) linked to shikimate *O*-hydroxycinnamoyltransferase-like (CST) and caffeoylshikimate esterase-like (CSE), with respectively FDR of 2.5E-9, 7.2E-47 and log2-FC of 1.719, 5.196, were overexpressed in response to UV-C treatment and *Xcv*.

#### Comparison of RNA-seq Analysis to qPCR

**FIGURE 5:**
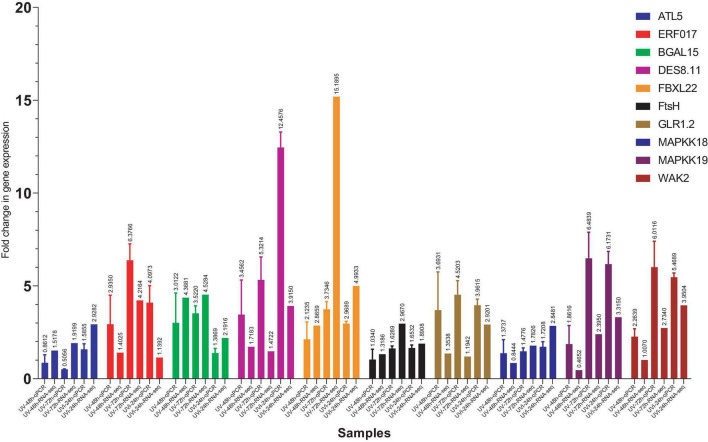
Comparison of gene expression levels between RNA-seq and qPCR. On the *X*-axis, we have qPCR data coupled to RNA-seq data for each gene and for each treatment: UV-48 h, UV after 48 h treatment; UV-72 h, UV after 72 h treatment; UVi-24 h, UV 24 h after inoculation. C-48h (Control corresponding to treated 48 h after UV) was used as a control.

Real-time quantitative polymerase chain reaction (qPCR) was performed on ten selected genes that were induced by the different treatments in our experimental conditions. Among these ten genes, *ATL5* and *ERF* are associated with defense mechanisms, *BGAL15*, *DES8.11*, and *FBXL22* are involved in plant growth recovery, *FtsH*, *GLR1.2*, *MAPKK18*, *MAPKK19*, and *WAK2* play a role in the return of homeostasis state following UV effects. All treatments were included in this validation, and all ten genes showed consistent trend of fold change expression between RNA-seq and qPCR (Figure 5).

Although the trend is similar for all tested genes (overexpression) for both PCR and RNSeq data, discrepancy in the amplitude of FCs can be observed for several genes. This is illustrated for example for ALT54 at UV-72 h (qPCR < RNA-seq), ERF017 at UV-48 h (qPCR > RNA-seq), ERF017 at UVi-24 h (qPCR > RNA-seq) and WAK2 at UV-72 h (qPCR > RNA-seq). At this point, we can only postulate that these apparent discrepancies are related to differences between the two techniques and handling error bias.

## Discussion

**FIGURE 6:**
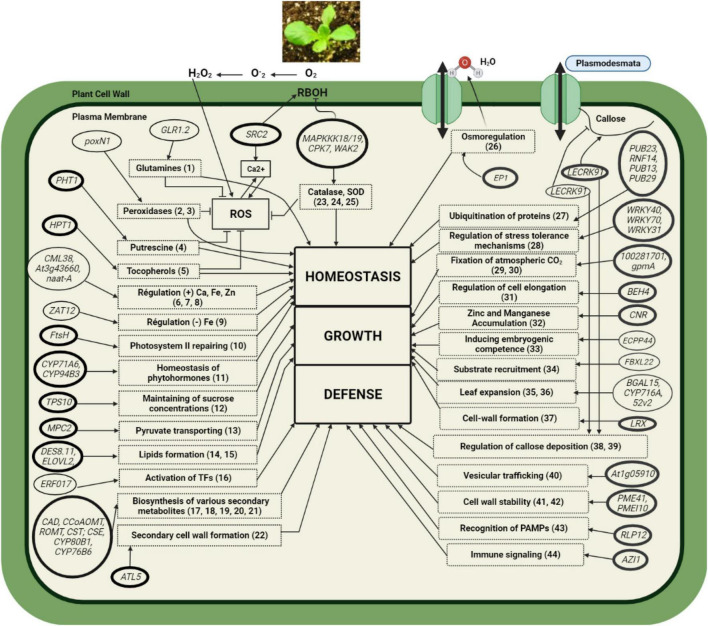
Representative model of the regulation mechanisms induced in lettuce by the preharvest application of hormetic doses of UV-C and inoculation with the pathogen *Xcv*. Thick-bordered rectangular boxes: Physiological (or biological) processes; dashed rectangular boxes: Function or molecules associated with genes as reported in the literature. Thin-bordered ellipses: genes affected by UV-C alone; thick-bordered ellipses: genes affected by UV-C + *Xcv* infection. The pointed arrows indicate activation reactions and the blunt arrows indicate inhibition reactions. The numbers in brackets in the rectangular boxes represent the reference source(s). (1) Cheng et al. (2018); (2) Kim et al. (2007); (3) Wang et al. (2016); (4) Pushpa et al. (2014); (5) Savidge et al. (2002); (6) Lokdarshi et al. (2016); (7) Gollhofer et al. (2014); (8) Cassin et al. (2009); (9) Le et al. (2016); (10) Kato and Sakamoto (2018); (11) Yan et al. (2016); (12) Lunn et al. (2014); (13) Shen et al. (2017); (14) Doan et al. (2009); (15) Nayeri and Yarizade (2014); (16) Dey and Vlot (2015); (17) Zhang and Liu (2015); (18) Ma et al. (2005); (19) Ziegler and Facchini (2008); (20) Sung et al. (2011); (21) Höfer et al. (2013); (22) Noda et al. (2013); (23) Zhou et al. (2014); (24) Bundó and Coca (2017); (25) Li et al. (2020); (26) van Engelen et al. (1993); (27) Kimura and Tanaka (2010); (28) Ashrafi-Dehkordi et al. (2018); (29) Hauser et al. (2015); (30) Li et al. (2018); (31) Chaiwanon et al. (2016); (32) Qiao et al. (2019); (33) Tan and Kamada (2000); (34) Skaar et al. (2013); (35) Ban et al. (2020); (36) Han et al. (2013); (37) Zhao et al. (2018); (38) Lannoo and Van Damme (2014); (39) De Storme and Geelen (2014); (40) Zhu et al. (2016); (41) Lionetti et al. (2017); (42) Niu and Wang (2020); (43) Jamieson et al. (2018); (44) Dutton et al. (2019).

This work confirms that a UV-C treatment of 1.6 kJ/m^2^ applied as described by Nicolas et al. (2020) falls within the hormetic dose range conferring a protective effect on lettuce against BLS. This is consistent with previous studies that have reported the efficacy of hermetic treatment of UV-C radiation in reducing the symptoms observed in a variety of plant-pathogen interactions (Janisiewicz et al., 2016a,b; Xu et al., 2019; Aarrouf and Urban, 2020; Nicolas et al., 2020; Vàsquez et al., 2020). Figure 6, which served as a framework for the discussion below, was developed using original data from the present study and available data from the literature. This figure shows that the early responses of lettuce treated with UV-C followed by *Xcv* inoculation led to a change in the transcriptome, which likely translated into a return to homeostasis, resumption of normal growth and the activation of defense mechanisms.

### Return to Homeostasis

The change in lettuce transcriptome demonstrated a return to the homeostasis state revealed by the relative overexpression of several genes. These genes act directly or indirectly on the production or removal of ROS to ensure the return and/or maintenance of homeostasis (Mittler et al., 2004).

Among those genes, the relative overexpression of *SRC2* genes and protein kinase biosynthesis genes (*MAPKK18*/*19*, *CPK7*, *WAK2*) was found. The *SRC2* genes induce the expression of NADPH oxidases, which are homologous proteins to respiratory burst oxidase homologs (RBOHs) (Kawarazaki et al., 2013). The latter are enzymes that contribute to the generation of ROS in response to hormonal and environmental signals (Suzuki et al., 2011). The *SRC2* genes encoding SRC2 proteins are involved in the activation of Ca^2+^ influx-dependent ROS generation in *Arabidopsis thaliana* (Kawarazaki et al., 2013). The relative overexpression of *SRC2* genes therefore suggests (Figure 6) ROS production. This would be in agreement with the work of Xu et al. (2014, 2019), who showed induction of ROS production in response to UV-C and bacterial flagellin, respectively.

In plants, RBOHs have an N-terminal cytosolic extension consisting of two Ca^2+^-binding EF-hand motifs and phosphorylation target sites that are important for their activity (Drerup et al., 2013). Considering the relative overexpression of SRC2, CML38 (calmodulin-like proteins), an increase in Ca^2+^ concentration can be presumed. Ca^2+^ is known to be essential to many plants cellular processes, including regulation of cellular ROS levels (Hu et al., 2018), division, differentiation, and programmed cell death (López-Fernández and Maldonado, 2015).

In contrast to SRC2, protein kinases negatively regulate RBOHD-dependent ROS production by, for instance, limiting the phosphorylation cascades that drive ROS production in response to stress (Escudero et al., 2019). Studies have shown that plants possess a comprehensive antioxidant system, consisting of enzymatic and non-enzymatic antioxidants, that can scavenge ROS (Baxter et al., 2014). Following the effect of UV-C treatment alone or in combination with *Xcv* inoculation, the relative overexpression of genes associated with the production of enzymatic antioxidants, such as SOD, CAT, and peroxidases (POD), and non-enzymatic antioxidants, such as tocopherols was found (Savidge et al., 2002). Tocopherols limit the oxidation of lipids to protect the thylakoid membrane from photo-oxidation. They also have antioxidant properties against oxidative damage caused by ROS. Our results also indicate an upregulation of metals such as Fe and Zn. Metal ions are cofactors of ROS detoxification enzymes (Ravet and Pilon, 2013). FtsH, an ATP-dependent metalloprotease with zinc as a cofactor, has been found to play a role in thylakoid membrane biogenesis, quality control in photosystem II repair, and the assembly of several protein complexes in photosynthetic electron transport pathways in *A. thaliana* (Kato and Sakamoto, 2018).

Our results indicate the relative overexpression of *PUB*, *WRKY*, and *EP1* genes with UV-C treatments alone or in combination with *Xcv* inoculation. *PUB* genes in several plant species are involved in the regulation of intracellular trafficking and ROS production (Tian et al., 2015) and in cell homeostasis (Kimura and Tanaka, 2010; Proietti et al., 2014). Genes associated with the hypothetical WRKY transcription factors participate in plant response to biotic (Scarpeci et al., 2013) and abiotic stresses (Scarpeci et al., 2013; Buti et al., 2019), by enabling dynamic cellular homeostatic reprogramming (Phukan et al., 2016). Studies have hypothesized that *EP1* genes may be involved in the regulation of water flow through the cell wall (van Engelen et al., 1993).

### Resumption of Normal Growth

In addition to the return and/or maintenance of homeostasis, the effect of UV-C treatments alone or in combination with *Xcv* inoculation was followed by a resumption of normal growth likely corresponding to an increase in photosynthetic activity. This increase in photosynthetic activity could generate an increase in dry matter synthesis (Farahbakhsh et al., 2017). Our recent work has also indicated an increase in dry matter in lettuce following preharvest treatments with hormetic doses of UV-C (Sidibé et al., 2021). In the present study, the effect of UV-C treatments alone or in combination with *Xcv* resulted in the relative overexpression of 13 genes directly or indirectly associated with the resumption of normal growth. Four of these genes (*ECPP44*, *BGAL15*, *FBXL22*, *CYP716A52v2*) were induced in response to the effect of UV-C alone. During somatic embryogenesis in plants, the phosphoprotein ECPP44 induces embryogenic competence in plants (Tan and Kamada, 2000). The induction of ECPP44 in response to the single effect of UV-C is therefore consistent with the overexpression of genes associated with embryogenic processes in cassava in response to drought (Wu et al., 2018). The β-galactosidases, such as BGAL15, are involved in polysaccharide metabolism (Ban et al., 2020) and in the separation of cellulose microfibrils during wall extension and remodeling (Sampedro et al., 2012). The released galactose fragments can serve as energy sources and substrates, which would explain the observed relative overexpression of the *FBXL22* gene. *FBXL22* is associated with F-box family proteins and is known to be involved in substrate recruitment (Skaar et al., 2013), plant growth, signal transduction (Huo et al., 2014), and disease tolerance (Kumar et al., 2020). The *CYP716A52v2* gene encodes the β-amyrin 28-oxidase enzyme, which catalyzes the triterpene hydroxylation responsible for the biosynthesis of oleic-type saponins (Han et al., 2013). Studies have shown that saponins and their biosynthetic intermediates are involved in plant growth and development (Moses et al., 2014) by stimulating chlorophyll biosynthesis, as observed in radish and pea (Boiteau and Ratsimamanga, 1956).

Seven genes (*DES8.11*, *TPS10*, *BEH4*, *MPC2*, *CNR*, and *LRX*) were induced in response to the joint effect of UV-C and *Xcv*. These seven genes code for proteins known to be involved in plant growth and development. The *DES8.11* gene is associated with fatty acid desaturase proteins, which at the plasma membrane site produce unsaturated fatty acids by introducing double bonds into fatty acid hydrocarbon chains (Nayeri and Yarizade, 2014). *DES8.11* gene induction is in agreement with the work of Gierz et al. (2017), who showed overexpression of fatty acid-encoding genes in algae (*Symbiodinium* sp.) in response to heat stress.

The *TPS* genes encode for a trehalose-6-phosphate synthase, which is a key enzyme for trehalose biosynthesis in yeast (Yeo et al., 2000). In beans (*Phaseolus vulgaris*), induction of trehalose-6-phosphate synthase biosynthesis has been shown to increase yield and improve drought tolerance (Suárez et al., 2008). BEH4 proteins of the BES1/BZR1 BES1 family integrate a variety of plant signaling pathways (Yin et al., 2005), such as brassinosteroid (BR) signaling. BRs are phytohormones involved in the regulation of cell elongation and lateral organ development (Chaiwanon et al., 2016). Pyruvate, as the end product of glycolysis, is derived from sources in the cell cytoplasm, and much of the pyruvate is transported into mitochondria by MPC2 proteins for oxidative metabolism of the Krebs cycle (Shen et al., 2017). Studies have shown that the *CNR* gene, which is relatively overexpressed in rice, has increased tolerance to otherwise toxic levels of Zn and Mn (Qiao et al., 2019). Mn has been shown to be an essential micronutrient for all stages of plant development, including as a catalytic center for photosystem II (Yang et al., 2007). The *LRX* gene is responsible for maintaining cell wall integrity through cross-linking with other cell wall components, such as pectin (Zhao et al., 2018). Overexpression of these genes (*TPS10*, *BEH4*, *MPC2*, *CNR*, and *LRX*) associated with cell tissue expansion is consistent with the increase in dry matter observed in lettuce following preharvest UV-C applications (Sidibé et al., 2021).

### Activation of Defense Mechanisms

The model in Figure 6 proposes an activation of defense mechanisms in lettuce via the relative overexpression of 15 genes following the effect of UV-C alone or in combination with *Xcv*. The effect of UV-C treatments alone resulted in the induction of the *ERF017* gene and the inhibition of the *LECRK91* gene. Genes associated with the structural reinforcement of the cell wall (*LECRK91*, *RLP12*, *ATL5*, *PMEI10*, *PME41*, *AZI1*, *At1g05910*, *CYP80B1*, *CYP76B6*, *PAL*, *CST*, and *CSE*) were induced by the effect of UV-C and *Xcv*. The induction of these genes suggests a “priming effect” of UV-C treatments in lettuce against the pathogen.

The inhibition of the *LECRK91* gene in response to the single effect of UV-C and its activation following the combined effect of UV-C and *Xcv* observed in the present study is not surprising. In *A. thaliana*, inhibition of *LECRK* gene expression has been established under various oxidative stresses (UV-B, drought, heat) (Mondal and Das, 2020). Conversely, overexpression of *LECRK* family genes was observed in response to infection by bacterial pathogens (Balagué et al., 2017). Receptor-like proteins (RLPs) often associate with receptor-like kinases (RLKs) and are involved in the development of immunity and growth (Jamieson et al., 2018). RLK proteins (Quezada et al., 2019) and Bcl-2-associated athanogenes (BAGs) (Williams et al., 2010) have been shown to cause cell death associated with a hypersensitivity reaction. For their part, the lectin receptor kinase (LECRK) and L-type lectin domain proteins (LLP) proteins (Hofberger et al., 2015) are pathogen recognition receptors (PRRs) involved in the recognition of carbohydrate structures of microbial organisms (Lannoo and Van Damme, 2014). The *ERF* gene induces the Ethylene-Responsive Factor (ERF) family of proteins, which in turn induce physiological responses to stress in plants (Dey and Vlot, 2015). Secondary wall biosynthesis in plants is induced by the *ATL5* gene (Noda et al., 2013). Also, it should be noted that the RING domain of ATL proteins is essential for E3 ubiquitin ligase (PUB) function in response to pathogen invasion (Deng et al., 2017). Therefore, it would be logical to assume that some complementarity between ATL and PUB proteins enabled the control of BLS observed in the present study.

Pectin methylesterase proteins can contribute to the generation of pectic fragments, some of which are reported to induce plant defense mechanisms (Wormit and Usadel, 2018). As PME activity is modulated by pectin methylesterase inhibitors (PMEI) (Wormit and Usadel, 2018), it is therefore appropriate to speculate that in this study, in conjunction with the relative overexpression of PME, the increased activity of PMEI10 contributes to protecting the lettuce cell wall from degradation by *Xcv*-secreted pectinases. It is well known that *Xcv* pectinases are important factors of virulence (Kazemi-Pour et al., 2004).

*AZI1* genes inducing pEARLI1-like lipid transfer protein biosynthesis link to glycerol-3-phosphate accumulation required for systemic acquired resistance conferred by azelaic acid (Yu et al., 2013). In rice plants, the AAA ATPase family protein-associated gene *At1g05910* is involved with the *PUB* genes in the induction of multivesicular body (MVB) trafficking of the plant defense system (Zhu et al., 2016). This result therefore suggests a complex feedback regulatory loop between AZI1, AAA ATPases, PUB, and glycerol-3-phosphate proteins.

In *A. thaliana*, the Geraniol 8-hydroxylase (G8H) (CYP76B6) biosynthetic gene have been shown to be involved in the flavonoid (Sung et al., 2011), alkaloid, and monoterpenoid biosynthetic pathway (Höfer et al., 2013). The precursors of G8H enzymes are derived from the deamination of L-phenylalanine (Baucher et al., 1998) which is catalyzed by PAL, the main entry point in the biosynthetic pathway of phenylpropanoids, including flavonoids, which play a role in pathogen defense and UV protection (Buer et al., 2010). Other enzymes, such as Caffeoyl shikimate esterase (CSE) and Shikimate *O*-hydroxycinnamoyltransferase-like (CST), which are involved in the lignin biosynthetic pathway (Vanholme et al., 2013), are relatively overexpressed in response to the effect of UV-C and *Xcv*. In addition to the overexpression of the latter two genes (*CSE* and *CST*), the overexpression of *ATL5* and *LECRK91* genes suggests that the protection of UV-C-treated lettuce would be partly due to the reinforcement of structural barriers. This UV-C effect was previously observed in tomato treated at the postharvest stage (Charles et al., 2008).

## Conclusion

The control of BLS following the application of the total UV-C treatment of 1.6 kJ/m^2^ observed in this study can be explained by a change in several physiological processes in lettuce. Transcriptome analysis of lettuce in response to UV-C alone or in combination with *Xcv* showed that the differentially expressed genes are associated with homeostasis, growth and defense. These results allow us to conclude that UV-C hormesis applied under the conditions described in the present study is an effective eustress that does not interfere with the ability of treated plants to resume normal growth or to defend themselves against potential stressors.

## Data Availability Statement

The original contributions presented in the study are publicly available in NCBI under accession number SUB10516369.

## Author Contributions

AS, MTC, and CB conceived and designed the study. AS performed the experiments. AS, MTC, J-FL, and YX analyzed the data. AS, MTC, and CB wrote the manuscript with valuable contributions from J-FL and YX. All authors read and approved the final manuscript.

## Conflict of Interest

The authors declare that the research was conducted in the absence of any commercial or financial relationships that could be construed as a potential conflict of interest.

## Publisher’s Note

All claims expressed in this article are solely those of the authors and do not necessarily represent those of their affiliated organizations, or those of the publisher, the editors and the reviewers. Any product that may be evaluated in this article, or claim that may be made by its manufacturer, is not guaranteed or endorsed by the publisher.

## Footnotes

1
https://bitbucket.org/mugqic/genpipes/src/master/
