# Supplementary material for: Preharvest UV-C Hormesis Induces Key Genes Associated With Homeostasis, Growth and Defense in Lettuce Inoculated With Xanthomonas campestris pv. vitians
Source: Front Plant Sci. 2022 Jan 17;12:793989. doi: 10.3389/fpls.2021.793989 (PMC8801786; doi:10.3389/fpls.2021.793989)
Supplement: Supplementary file 1 [file Data_Sheet_1.docx]

Supplementary Material


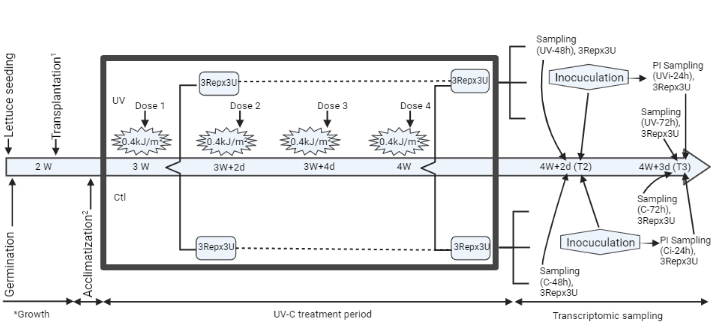


**Figure S1.** UV treatment and sampling schedule.

The central line represents the timeline of one experiment.

* Lettuces were grown for 2 weeks (2W) before transplantation.

^1^ Transplantation was done when the lettuce plants were at the 4- to 5-leaf stage.

^2^ Acclimatization lasted a week before the beginning of the UV treatment.

Ctl : Control group; UV : UV-C group; UV-C treatment started 3 weeks (3W) after seeding.

3W+2d : 3 weeks and 2 days; Inoculation was performed 2 days after application of the 4^th^ dose at 4W+2d; PI : Post Inoculation with *Xcv*.

3Repx3U: three repetitions of three experimental units (1U= unit of lettuce plant per pot)


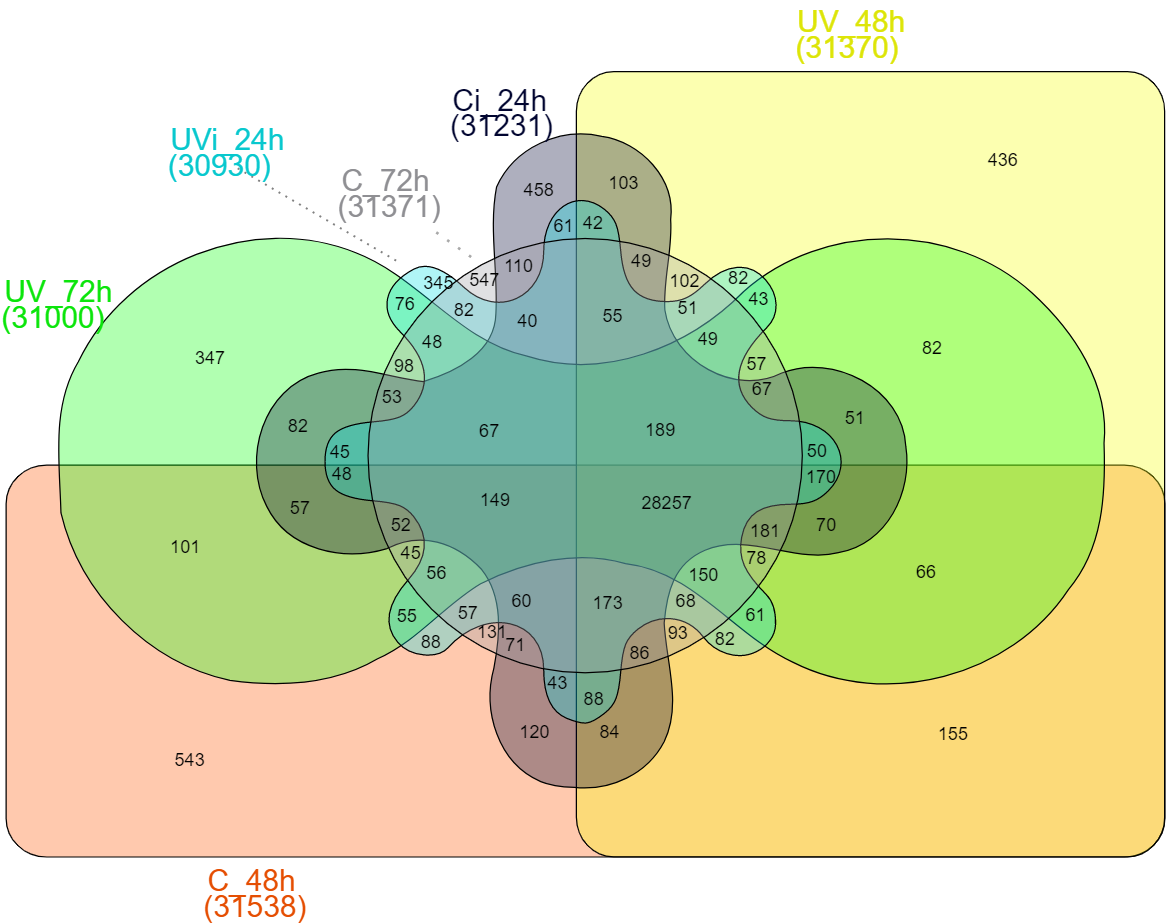


**Supplementary Figure S2.** Venn diagram according to the different treatment groups and sampling time of the 46150 transcripts obtained. C_48h: lettuces corresponding to lettuces treated 48 h after the last UV treatment. UV_48h: treated lettuces, 48h after the last UV treatment. C_72h: control lettuces corresponding to treated lettuces 72h after the last UV treatment. UV_72h: treated lettuces, 72h after the last UV treatment. Ci_24h: control and inoculated lettuces, corresponding to treated lettuces 24 h after inoculation. UVi_24h: treated and inoculated lettuces, 24 h after inoculation.


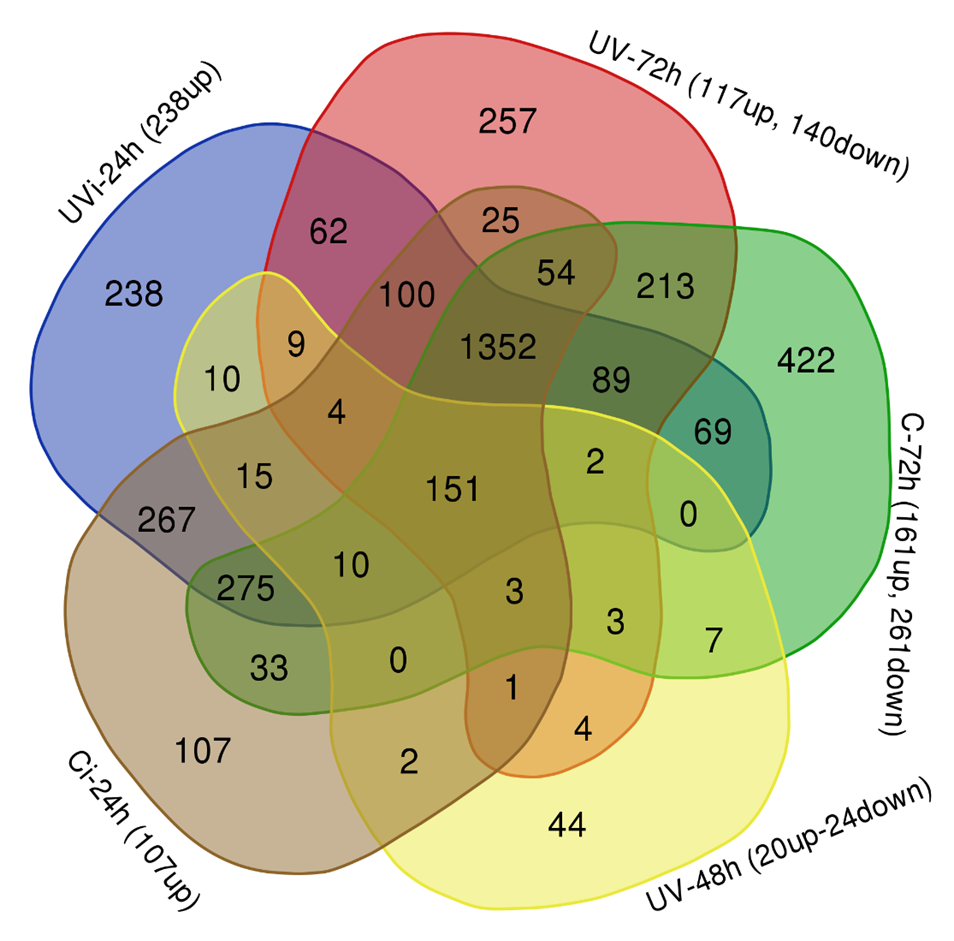


**Figure S3.** Venn diagram of differentially expressed genes in lettuce (|log2-FC| > 1.5 and FDR < 0.05) after UV-C treatments and *Xcv* inoculation. UV-48h: UV after 48h treatment, C-72h: Control corresponding to treated samples 72 h after UV; UV-72h: UV after 72 h treatment; Ci-24h: Control 24 h after inoculation; UVi-24h: UV 24 h after inoculation. http://bioinformatics.psb.ugent.be/webtools/Venn/.

**Supplementary Table S1.** Experimental design for transcriptomic study.

| **Sample Description** | **Identification in datasheet** | **Replicates^1^** | **Experiments^2^** |
| --- | --- | --- | --- |
| Control 48h (corresponding control for UV after 48h) | E1_D0_T2 | 03-janv | E1,E2,E3 |
| UV after 48h treatment | E1_D2_T2 | 03-janv | E1,E2,E3 |
| Control 72h (corresponding control for UV after 72h) | E1_D0_T3 | 03-janv | E1,E2,E3 |
| UV after 72h treatment | E1_D2_T3 | 03-janv | E1,E2,E3 |
| Control 24h after inoculation^3^ | E1_D0i_T3 | 03-janv | E1,E2,E3 |
| UV 24h after inoculation^3^ | E1_D2i_T3 | 03-janv | E1,E2,E3 |

^1^There were 3 replicates for each sample

^2^Three (3) independent experiments were conducted (E1, E2, E3)

^3^Inoculation was performed 48h after UV treatment in both UV treated and corresponding control

**Supplementary Table S2.** List of selected primers.

| **Gene ID** | **Symbol** | **Name** | **Primers** | **Start** | **Stop** | **Length** | **Tm** | **GC%** |
| --- | --- | --- | --- | --- | --- | --- | --- | --- |
| **18254** | LOC111913257 | ATL54 |  |  |  |  |  |  |
|  |  |  | CAATCGCTCCACGGAAGAA (Sense) | 1286 | 1305 | 19 | 62 | 53 |
|  |  |  |  |  |  |  |  |  |
|  |  |  | GTCCTGCTGGTATGAGTTTAGG (AntiSense) | 1370 | 1392 | 22 | 62 | 50 |
| **1159** | LOC111905898 | ERF017 |  |  |  |  |  |  |
|  |  |  | AACTCCACCCTTCCCAGATA (Sense) | 331 | 351 | 20 | 62 | 50 |
|  |  |  |  |  |  |  |  |  |
|  |  |  | CCTGCAACTGAGACGATGAA (AntiSense) | 410 | 430 | 20 | 62 | 50 |
| **38054** | LOC111889078 | DES8.11 |  |  |  |  |  |  |
|  |  |  | CTAGCATCCTCACTGGCTTATG (Sense) | 470 | 492 | 22 | 62 | 50 |
|  |  |  |  |  |  |  |  |  |
|  |  |  | GTGGAGGATAAAGCCGATAGTG (AntiSense) | 549 | 571 | 22 | 62 | 50 |
| **45323** | LOC111897257 | FBXL22 |  |  |  |  |  |  |
|  |  |  | ATCCAAGCGATCAAAGGTATCA (Sense) | 129 | 151 | 22 | 62 | 41 |
|  |  |  |  |  |  |  |  |  |
|  |  |  | GTGGATGGAAGACGTGAAAGA (AntiSense) | 210 | 231 | 21 | 62 | 48 |
| **29845** | LOC111879998 | BGAL15 |  |  |  |  |  |  |
|  |  |  | GGCTATCCTCACCCAAGAATG (Sense) | 4432 | 4453 | 21 | 62 | 52 |
|  |  |  |  |  |  |  |  |  |
|  |  |  | TCCGCTGCTCCAAACTTATC (AntiSense) | 4490 | 4510 | 20 | 62 | 50 |
| **20758** | LOC111916025 | FtsH |  |  |  |  |  |  |
|  |  |  | GCTTCTCGTGTGAGGGATTT (Sense) | 998 | 1018 | 20 | 62 | 50 |
|  |  |  |  |  |  |  |  |  |
|  |  |  | CAGTTCCTCTCTGTCTTCCAAC (AntiSense) | 1076 | 1098 | 22 | 62 | 50 |
| **34376** | LOC111884950 | MAPKKK18 |  |  |  |  |  |  |
|  |  |  | CCTTCACGTTTGCCCATTTG (Sense) | 996 | 1016 | 20 | 62 | 50 |
|  |  |  |  |  |  |  |  |  |
|  |  |  | CGACTCAGTGTTGTCCTCTTC (AntiSense) | 1074 | 1095 | 21 | 62 | 52 |
| **23556** | LOC111919099 | MAPKKK19 |  |  |  |  |  |  |
|  |  |  | AGGGAGATAGTTCGAGGGTTAG (Sense) | 652 | 674 | 22 | 62 | 50 |
|  |  |  |  |  |  |  |  |  |
|  |  |  | TCGCACCACTTTCGTCAAT (AntiSense) | 730 | 749 | 19 | 62 | 47 |
| **34502** | LOC111885144 | WAK2 |  |  |  |  |  |  |
|  |  |  | GGGTCAAGGCACAGTGTATAA (Sense) | 1994 | 2015 | 21 | 62 | 48 |
|  |  |  |  |  |  |  |  |  |
|  |  |  | ATTGCTCTAGTTGGCTCTCATC (AntiSense) | 2067 | 2089 | 22 | 62 | 46 |
| **2086** | LOC111915435 | GLR1.2 |  |  |  |  |  |  |
|  |  |  | CGACAATGGTGAGAAGAGAGTAG (Sense) | 1476 | 1499 | 23 | 62 | 48 |
|  |  |  |  |  |  |  |  |  |
|  |  |  | AGCTTCAGGACCTAGAGTAGAG (AntiSense) | 1563 | 1585 | 22 | 62 | 50 |

**Supplementary Table S3.** Functions of interest genes presented in the results and the associated reference(s) in the literature. <https://figshare.com/s/1c3b3be2716d6f4798b1>

**Supplementary Table S4.** RNA-Seq Reads and Mapping. <https://figshare.com/s/19ff6583babffd6e19b3>

**Supplementary Table S5.** Genes found by sample after comparison with sample control (C-48h). The genes were filtered out with a threshold |log2-FC| > 1.5 and FDR < 0.05. <https://figshare.com/s/4fc4ed8793a276cf185f>

C-48h: Control corresponding to treated samples 48h after the last UV treatment dose application. UV-48h: UV after 48h treatment, C-72h: Control corresponding to treated 72h after UV, UV-72h: UV after 72h treatment, Ci-24h: Control 24h after inoculation, UVi-24h : UV 24h after inoculation. The genes were filtered out with a threshold |log2-FC| > 1.5 and FDR < 0.05.
